# Complete Genome Sequence of Human Adenovirus Type 7 Associated with Fatal Infant Pneumonia

**DOI:** 10.1128/genomeA.00182-12

**Published:** 2013-02-14

**Authors:** Liuying Tang, Junjing An, Pengbo Yu, Wenbo Xu

**Affiliations:** a National Institute for Viral Disease Control and Prevention, Chinese Centre for Disease Control and Prevention, Beijing, People’s Republic of China; b Fengtai District, Beijing Center for Disease Control and Prevention, Beijing, People’s Republic of China; c Shaanxi Center for Disease Control and Prevention, Xi’an, People’s Republic of China

## Abstract

The Chinese human adenovirus 7 (HAdV7) 0901HZ/ShX/CHN/2009 was isolated from the hydrothorax fluid of an infant with fatal pneumonia in Shaanxi, China, in 2009. Comparison of the entire genome with the genomes of the other 10 strains of HAdV7 from GenBank revealed homologies of 89.9 to 99.9%, with geographic polymorphism among HAdV-7 field strains circulating in mainland China.

## GENOME ANNOUNCEMENT

Human adenoviruses (HAdVs) can cause a broad range of clinical manifestations in humans, with billions of people infected worldwide (1). These adenoviruses are grouped into seven species (A to G), and to date fifty-six types (HAdV-1 to HAdV-56) have been identified on the basis of nucleotide and deduced amino acid sequences (2, 3). The increasing application of genomics and bioinformatics approaches to the investigation of HAdV genomes will lead to an understanding of the emergence of novel HAdV pathogens, the identification and performance of surveillance for these pathogens, and perhaps the control of epidemic outbreaks with effective and appropriate vaccines (4).

An outbreak of infant severe pneumonia was reported in Shaanxi, China, in 2009 (5), and two adenovirus 7 strains were isolated from the hydrothorax fluid of the patients. For one strain, named 0901HZ/ShX/CHN/2009 (HAdV7-0901HZ), sequencing and analysis of the whole genome were conducted. For genome sequencing, a standard PCR was conducted by using a previously described procedure (6). Raw sequence data were assembled using Sequencher software (version 4.0.5).

Pipmaker (http://bio.cse.psu.edu/pipmaker) (7) was used to perform nucleotide dot blot identity-based analyses of the whole HAdV genomes now available from GenBank. A total of 10 complete genome sequences of HAdV7 (not including the one in this study) are available in the GenBank database, including the prototype type, vaccine strains, and field strains, of which three were isolated from the south of China, Guangzhou, and Chongqing. The strain 0901HZ was isolated from the northwest of China, in the Shaanxi Province.

The strain HAdV7-0901HZ has a genome of 35,239 bp in length and a GC content of 51.08%. A total of 48 coding sequences were identified in this genome sequence, including seven predicted genes.

The genome sequence alignment of HAdV7-0901HZ with other HAdV7 strains revealed high homologies of 99.7 to 99.9% (accession numbers JX625134, JN860677, and GQ478341). An exception was HAdV7-GZ07, which has a large fragment deletion at position 28334 to 31162 of the E3 region (accession number HQ659699) and homology of 89.9%. For the vaccine and Gomen strains (accession nos. AY594255 and AY594256, respectively) the nucleotide sequence similarities were 97.7% and 99.4%, respectively

Adenovirus infections in the immunocompetent host are usually limited by the immune system. But HAdV7, a member of the B1 subspecies, has been identified in epidemics to be highly virulent and is associated with clinical manifestations of considerable severity, including residual lung damage and fatal outcomes (8–10). This pathogen has been found in outbreaks in the United States and Asia and has become a global concern (11–13). It also poses a special threat for infants and young children as their immune systems are not completely established.

Comparative genomic analysis of 11 strains of human adenovirus type 7 isolated from this study and the references from GenBank showed that HAdV-7 circulating in mainland China has multiple transmission chains, suggesting the need for surveillance in high-risk populations.

### Nucleotide sequence accession number.

The nucleotide sequence of the complete genome of HAdV7 strain 0901HZ/ShX/CHN/2009 has been submitted to GenBank under accession number JF800905.
